# Macular buckling alone versus combined inverted ILM flap on macular hole-associated macular detachment in patients with high myopia

**DOI:** 10.1038/s41433-023-02406-1

**Published:** 2023-01-31

**Authors:** Xiujuan Zhao, Huiying Song, Silvia Tanumiharjo, Yanbing Wang, Yuqing Chen, Shida Chen, Xia Huang, Bingqian Liu, Ping Lian, Lin Lu

**Affiliations:** State Key Laboratory of Ophthalmology, Zhongshan Ophthalmic Center, Guangdong Provincial Key Laboratory of Ophthalmology and Visual Science, Guangdong Provincial Clinical Research Center for Ocular Diseases, Sun Yat-sen University, Guangzhou, 510060 China

**Keywords:** Outcomes research, Eye diseases

## Abstract

**Purpose:**

To compare the efficacy of macular buckling (MB) alone against a combined internal limiting membrane (ILM) inversion flap for full-thickness macular hole (FTMH)-associated macular detachment (MD) in patients with high myopia.

**Methods:**

This was a prospective interventional case series of patients with high myopia surgically treated with MB alone or combined with an inverted ILM flap for FTMH- associated MD. Best-corrected visual acuity (BCVA) at the 24-month postoperative follow-up, rate of initial retinal reattachment and macular hole closure were measured.

**Results:**

A total of 62 eyes from 62 participants (33 in the MB group, 29 in the combination group) were studied. Postoperative BCVA improved significantly in both the combination group (*P* < 0.001) and the MB group (*P* = 0.027). The postoperative BCVA at 12 months (*P* = 0.021) and 24 months (*P* = 0.041) was significantly better in the combination group than in the MB group. The postoperative BCVA was not significantly different between the eyes with closed and unclosed MH at each follow-up time point (*P* > 0.05). In the combination group, we observed earlier retinal reattachment and closure of the MH as well as a higher rate of MH closure (82.8% vs. 66.7%) than in the MB group, although this difference was insignificant (*P* = 0.248).

**Conclusion:**

MB combined with the ILM flap inversion technique achieved better postoperative BCVA and a higher success rate of MH closure than MB alone. We believe that combination surgery should be preferentially recommended.

## Introduction

Macular detachment (MD) associated with a full-thickness macular hole (FTMH) is a severe vision-threatening complication of high myopia [1]. High myopia patients with FTMH require urgent surgical intervention because they can easily develop to retinal and choroidal detachment. Several surgical methods have been attempted to treat high myopia patients with FTMH, including pars plana vitrectomy (PPV) with internal limiting membrane (ILM) peeling [2] or ILM flap inversion [3–7], lens capsular flap transplantation [8], autologous serum injection [9], autologous neurosensory retinal flap transplantation [10], human amniotic membrane grafting [11], and gas or silicone oil tamponade. Previous studies demonstrated that the inverted ILM flap technique can provide a bridge that helps the edges of the macular hole to migrate centripetally, thus achieving a better functional and anatomical prognosis, especially in large macular holes (MHs) [12], in high myopia MHs, and in refractory MHs [13, 14]. However, the MH is often accompanied by posterior staphyloma and expanded eyeball in eyes with high myopia, PPV alone often caused more severe macular atrophy progression after surgery than MB [11, 15], and primary anatomical success was significantly lower when compared to emmetropic patients [16].

Macular buckling (MB) has shown success in the treatment of MH-associated MD in patients with high myopia, as MB corrects posterior staphyloma, thus releasing anteroposterior traction forces and aiding reattachment [17, 18]. Our previous study showed that the retinal reattachment success rate was significantly higher with MB than with PPV, although there was no significant difference in the MH closure rate [15]. Due to the complexity of the macular hole in high myopia, combined surgery is recommended.

Thus, our study aims to evaluate and compare the long-term effects of MB alone to combined ILM flap inversion for high myopia patients with MH-associated MD. Our results can provide evidence to improve surgical design and obtain more satisfactory results in the surgical management of high myopia patients with MH-associated MD.

## Methods

### Trial design

This prospective study was designed to explore the prognosis of high myopia patients with full-thickness macular hole (FTMH)-associated macular detachment (MD) undergoing the macular buckling (MB) surgery alone (the MB group) or combined with an internal limiting membrane (ILM) inversion flap (the combination group). High myopia patients with FTMH-associated MD due to axial length elongation were recruited between January 2017 and November 2019 at Zhongshan Ophthalmic Center in Guangzhou, China. Randomized grouping was conducted after participants were informed about the purpose of this study and informed consent was signed. This study was approved by the Ethical Review Committee of Zhongshan Ophthalmic Center and adhered to the tenets of the Declaration of Helsinki.

It was assumed that MH closure rate would be 64% after receiving MB and 90% after receiving combined surgery, with 80% statistical power and a two-sided test. Thus, to allow for a 3% loss during follow-up, a total of 70 participants were required to participate in this study, with 35 patients in each arm (calculated using G-Power V.3.1.9.2 software).

### Inclusion and exclusion criteria

High myopia patients between the age of 18 and 70 years old, with an axial length (AL) longer than 26.5 mm or a refractive error (spherical equivalent) less than −8.0 dioptres, and presence of FTMH-associated MD on optical coherence tomography (OCT) were included. Our exclusion criteria were patients with a history of ocular trauma or glaucoma, an active intraocular haemorrhage or inflammation, any media opacity that precluded imaging or clinical evaluation of the macula, a history of intraocular surgery except cataract surgery, and retinal detachment extending beyond the vascular arcades.

### Participants

**Fig. 1:**
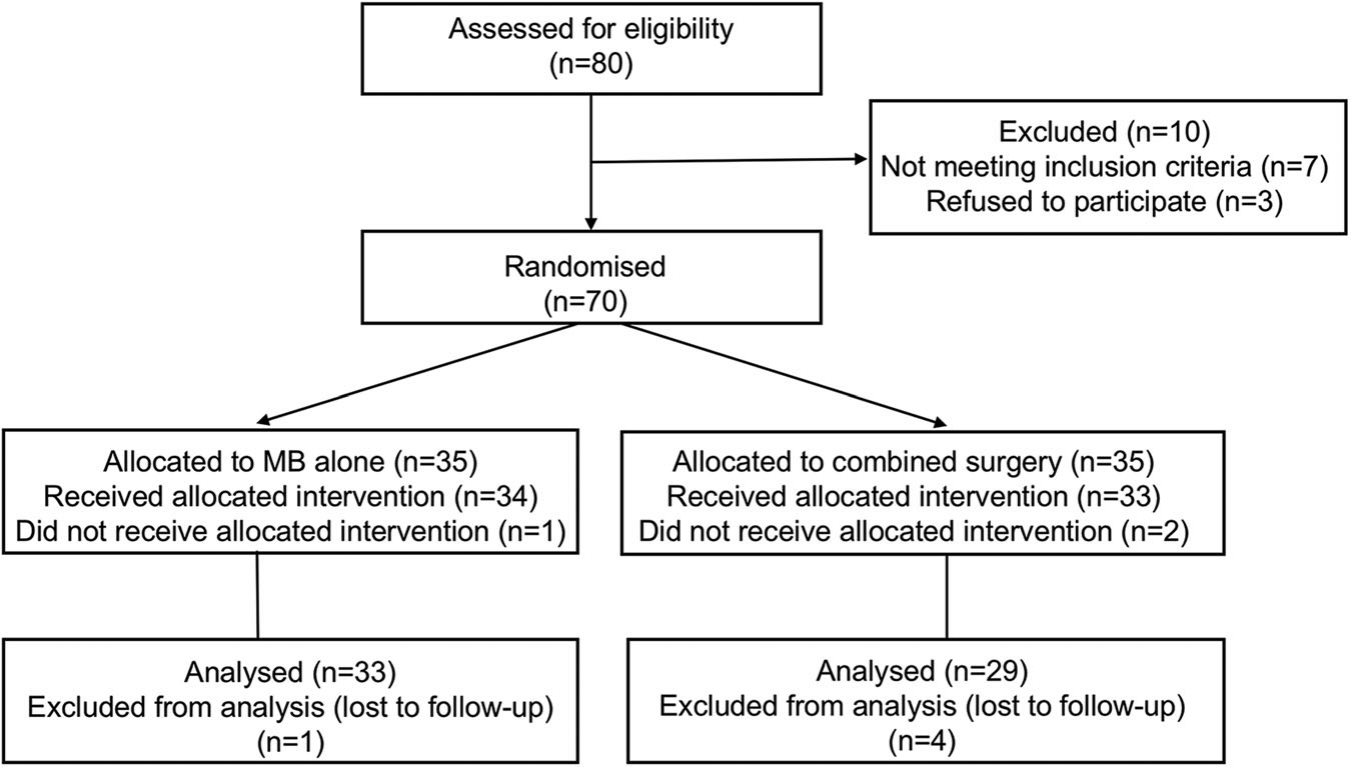
Consort diagram. Flowchart of randomization and follow-up for the study.

A total of 80 high myopia patients with FTMH-associated MD on OCT images were initially recruited, of which 7 patients were excluded according to the exclusion criteria that had extended retinal detachment beyond the vascular arcades while awaiting surgery, 3 patients declined participation. A total of 70 patients were finally included, with randomized grouping of 35 eyes in the MB group and 35 eyes in the combination group (Fig. 1).

### Randomization

The 70 participants included were randomized according to a 1:1 ratio using a random number generator and assigned to undergo either MB alone surgery or MB combined with ILM flap inversion. The patients were given a detailed explanation of their assigned surgical procedures and understood the risks of potential complications before giving consent.

### Surgical technique

One experienced vitreoretinal surgeon (LL) performed all the surgeries under retrobulbar or general anaesthesia. The MB surgery was performed using a silicone sponge-titanium exoplant, as described in our previous studies [15, 19]. Under general or retrobulbar anaesthesia, the surgery began with a 360^o^ limbal periotomy. The superior rectus, lateral rectus, inferior rectus and the inferior oblique muscle were separated from the surrounding tendons. The rectus muscles were then looped using silk ties to allow for rotation of eye position. One of the distal ends of the silicone band was passed under the inferior oblique muscle, lateral rectus and inferior rectus, then it was sutured to the sclera on the nasal side of the inferior rectus muscle using a spatulated needle with 5-0 non-absorbable thread (Ethicon, Johnson & Johnson Medical Ltd, China) approximately 12 mm posterior to the limbus. The other end of the band crossed the superior rectus muscle, and it was sutured to the sclera on the nasal side of the superior rectus muscle. Release part of the aqueous humour by anterior chamber puncture. The sponge-enveloped titanium plate was sutured in the superior temporal quadrant. The length of the upper and lower bands and the location of the temporal titanium plate sutures were calculated from preoperative 3D MRI of patients. Intraoperative adjustments were made under indirect fundoscopy to ensure that the surgical ridge was located in the centre of the macula.

The participants in the combined group underwent MB, followed by 25-gauge PPV. A 25-gauge PPV was performed according to a regular procedure. The core and posterior hyaloid were removed, followed by a first air-fluid exchange drained subretinal fluid (SRF), which temporarily reattached the retina. Indocyanine green was used to stain the ILM, which was then peeled using microforceps. The ILM flap was gently inverted to cover the MH, and perfluorocarbon liquid (PFCL) was injected to stabilize the ILM flap. Air-fluid exchange and air-PFCL exchange were then conducted, eventually filling the vitreous cavity with filtered air. At the end of the surgery, viscoelastic agents were dropped upon the ILM flap to avoid flap displacement. Patients were instructed to maintain a prone position for 3 days after surgery.

### Outcome measurements

All participants returned for follow-up at 6 months, 12 months and 24 months postsurgery for evaluation. The outcome parameters assessed were best corrected visual acuity (BCVA), intraocular pressure (IOP) measurements (TX-200 tonometer; Canon, Japan), AL measurements (IOL Master; Carl Zeiss, Germany), OCT (Heidelberg Engineering, Heidelberg, Germany; DRI-OCT, Topcon Corp, Tokyo, Japan), retinal reattachment, MH closure and complications. The absence of a foveal defect and absence of bare RPE exposure to the vitreous were regarded as MH closure. The technician who performed the examination was blind to the patients’ group.

### Statistical analysis

Data processing and analysis were performed with SPSS software for Windows (V23.0). Data are presented as the mean ± SD. For variables with a normal distribution, 2 independent t tests were used for comparison. For variables with a nonnormal distribution, Mann-Whitney U tests were performed for analysis. Chi-square tests were used to assess qualitative data. One-way analysis of variance was conducted to analyse presurgery and postsurgery BCVA and AL. The results were considered statistically significant when the *P* value was less than 0.05.

## Results

A randomized grouping of 70 eyes from 70 participants included had 35 participants in the MB surgery group and 35 participants in the combined surgery group. One participant in the MB group and two participants in the combination group did not receive their allocated intervention. Due to incomplete postsurgical follow-up, 1 participant in the MB group and 4 participants in the combined group were excluded from the final analysis. Patient demographics and ocular characteristics between the two groups were well matched (Table 1).

**Table 1:** Clinical characteristics and BCVA of the highly myopic patients of macular hole associated macular detachment who underwent MB or combined surgery.

	MB	Combination	*P*
NO. of eyes	33	29	
Sex (M: F)	6: 27	5: 24	0.923
Age (Y)	61.45 ± 8.33	57.83 ± 10.15	0.128
AL (mm)	29.77 ± 1.49	29.62 ± 1.90	0.734
IOP (mmHg)	14.53 ± 3.01	13.70 ± 2.69	0.263
BCVA (logMAR)	1.32 ± 0.50	1.23 ± 0.41	0.437
Aphakic eye /			
Intraocular lens eye /	0/4/29	0/5/24	0.834
Phakic eye			
AMM			
A1	2	0	
A2	24	19	
A3	7	10	
A4	0	0	
BCVA			
Pre-op	1.32 ± 0.50	1.23 ± 0.41	0.437
6 months	1.04 ± 0.51	0.88 ± 0.33	0.132
12 months	1.05 ± 0.50	0.81 ± 0.30	0.021
24 months	0.98 ± 0.46	0.77 ± 0.29	0.041
*P*	0.027	< 0.001	

*MB* Macular buckling, *AL* Axial length, *IOP* Intraocular pressure, *BCVA* Best-corrected visual acuity, *logMAR* Logarithm of the minimum angle of resolution, *AMM* Atrophic myopic maculopathy.

### Comparison of BCVA between the two groups

After surgery, there was significant improvement in BCVA for both groups (*P* < 0.05). The BCVA was significantly better in the combination group at 12 months (*P* = 0.021) and 24 months (*P* = 0.041) than in the MB group, but not at 6 months (*P* = 0.132) (Table 1). For eyes with MH closure, there was significant improvement in BCVA in the combination group (*P* < 0.001); however, this improvement was not significant in the MB group (*P* = 0.058). For eyes without MH closure, there was no significant improvement in BCVA for either group (*P* > 0.05). Comparing eyes with and without MH closure, BCVA was not significantly different at every follow-up time point within both groups (*P* > 0.05) (Table 2).

**Table 2:** Comparison of the BCVA (logMAR) between closed and unclosed MH in the two groups.

Group		Closed MH	Unclosed MH	*P*
MB	Pre-op	1.32 ± 0.49	1.32 ± 0.53	0.985
	6 months	1.00 ± 0.55	1.13 ± 0.41	0.528
	12 months	1.01 ± 0.52	1.14 ± 0.46	0.288
	24 months	0.96 ± 0.47	1.01 ± 0.45	0.762
	*P*	0.058	0.575	
Combination	Pre-op	1.26 ± 0.43	1.09 ± 0.29	0.403
	6 months	0.87 ± 0.33	0.92 ± 0.36	0.752
	12 months	0.81 ± 0.30	0.80 ± 0.31	0.768
	24 months	0.77 ± 0.29	0.80 ± 0.31	0.618
	*P*	< 0.001	0.628	

*BCVA* Best-corrected visual acuity, *MH* Macular hole, *MB* Macular buckling.

### Comparison of retinal reattachment and MH closure rates between the two groups

Retinal reattachment was achieved in 32 (32/33, 97.0%) eyes in the MB group and 29 (29/29 = 100%) eyes in the combination group at 24 months postoperatively. There was 1 eye in the MB group in which neither retinal reattachment nor MH closure was achieved during the follow-up period. MH closure was reached in 22 (22/33, 66.7%) eyes in the MB group and in 24 (24/29, 82.8%) eyes in the combination group at 24 months postoperatively.

**Fig. 2:**
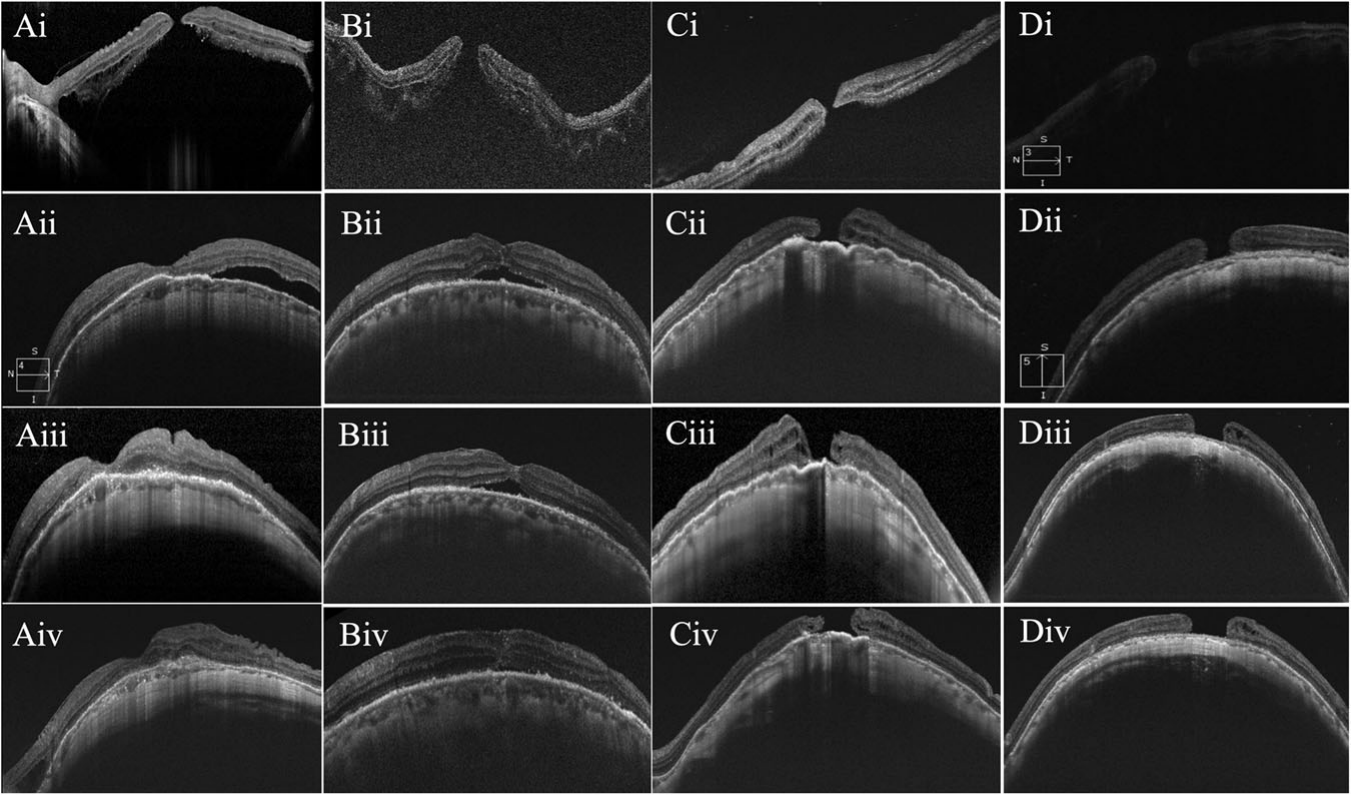
Optical coherence tomography images of patients with successful macular hole (MH) closure and without MH closure after macular buckling (MB) or combined surgery. **Ai** Presurgical image of MH-associated macular detachment (MD) from a patient in the MB group. **Aii** Closed MH and residual subretinal fluid (SRF) outside the fovea 3 months after MB surgery. **Aiii**–**iv** Retinal reattachment with MH closure at 1 year and 2 years after MB. **Bi** A presurgical image of MH and MD of a patient from the combination group. **Bii** MH closure with MD at 1 month after combined surgery. **Biii** Image showing most of the SRF absorbed at 6 months after surgery. **Biv** Retinal reattachment with MH closure at 18 months after surgery. **Ci** Presurgical image of MH and macular detachment (MD) from a patient in the MB group. **Cii**–**iv** Images of retinal reattachment with remaining unclosed MHs at 1 month, 6 months and 2 years after surgery. **Di** A presurgical image of a patient in the combination group with MH and MD. **Dii**–**iv** Images of retinal reattachment without MH closure at the 3-month, 12-month and 24-month follow-ups after combined surgery.

A higher rate of retinal reattachment was found in the combination group at one month after surgery (*P* = 0.018), but no difference was found between the two groups at 3 months, 6 months, 12 months and 24 months after surgery. The rate of MH closure was higher in the combination group at one month (*P* < 0.001) and 3 months (*P* = 0.04), but the difference in rate was not significant at 6 months, 12 months, and 24 months postsurgery (Supplementary Table 1) (Fig. 2).

In all the eyes of the combination group, ILM flap inversion was attempted. However, successful inversion was achieved in only 20 (20/29 = 69.0%) eyes. For these 20 eyes with successful ILM flap inversion, the MH closure rate was 100%. However, the MH failed to close in five of the nine eyes where ILM flap inversion was unsuccessful (Fig. 2).

### Comparison of axial length between the two groups

There was no significant difference in AL between the two groups at any time point (*P* > 0.05). After surgery, AL was significantly shortened for both groups (*P* < 0.001). There was also a trend of gradual regression of AL in both groups up to 12 months postsurgery (Supplementary Table 2).

### Complications after surgery

**Fig. 3:**
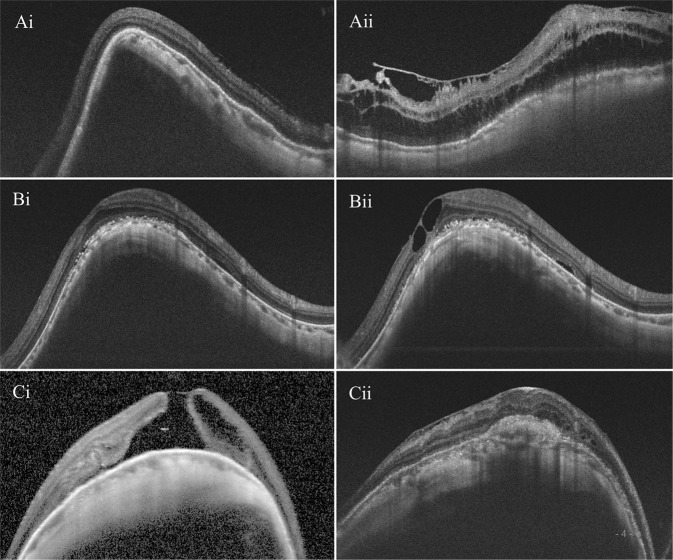
Postoperative complications seen on optical coherence tomography. **Ai** The image at 6 months after MB group shows the retinal reattachment with MH closure. **Aii** The image at postoperative year 1 shows the retinoschisis with an obvious decrease in the surgical ridge. **Bi** The image at 1 year after combination surgery shows retinal reattachment with MH closure. **Bii** Image of intraretinal cysts at 23 months after surgery. **Ci** The image at 1 month after MB surgery showing MH unclosure without CNV. **Cii** The image at 1 year after surgery showing MH closure with choroidal neovascularization.

Diplopia and metamorphopsia were the most common postoperative complications (65/67, 97.01%) in the early postoperative period, and these symptoms tended to recover or decrease over time. Within the 24-month follow-up period, cataract surgery was performed for 5 patients in the MB group and 9 patients in the combination group. There were 2 eyes in the MB group that developed retinoschisis after retinal reattachment and MH closure with disappearance of the surgical ridge. Intraretinal cysts were observed in three cases from the MB group and in two cases from the combination group. Choroidal neovascularization (CNV) was observed in one eye from the MB group after surgery (Fig. 3).

## Discussion

MH with retinal detachment in patients with high myopia has been difficult to manage and poses great challenges for vitreoretinal surgeons. This study compared the effect of MB and combined surgery with ILM flap inversion on FTMH- associated MD. All patients in the combined group were treated with MB, PPV, ILM flap inversion and filtered air as tamponade. Overall, the combined surgery achieved a higher and earlier MH closure, and BCVA improved for both groups, although MH closure did not account for a significant difference in postsurgical BCVA.

Several techniques have been proposed to treat high myopia MH. The autologous retinal transplantation technique to repair high myopia MH was studied by Moysidis et al. [20] and a high rate of hyperreflective foci was found in the graft early after surgery. On the other hand, Chen et al. [21] reported that excessive gliosis was observed with an inverted ILM flap. Gliosis is necessary for MH closure, a process during which neurotrophic factors and basic fibroblast growth factors are produced by activated Müller cells, and the ILM helps in cocultured Müller cell proliferation [22]. Eyes with high myopia have a much thinner ILM [23, 24], and as a response to the elongated globe in the eye with high myopia, Müller cells enter the ILM, causing Müller cell debris to accumulate, thus providing a damage-repairing microenvironment that promotes glial proliferation [23]. However, excessive gliosis can be toxic to neurons. The resulting irregular surface of the retina caused by excessive gliosis could lead to metamorphopsia.

In our study, the MH closure rate was 82.8% in the combination group and only 66.7% in the MB group. The BCVA was better in the combined group at 12 months and 24 months postoperatively than that in the MB group. However, the BCVA between closed MH and unclosed MH was not significantly different, which was in line with other studies. [13, 21, 25]

Previous studies have shown that ELM and EZ restoration was associated with improvement of postsurgical BCVA [26]. However, the outer retinal layer was difficult to identify in our study due to chorioretinal atrophy, indicating that irreversible damage had occurred in the photoreceptor layer. In a regular macular hole, closure was facilitated by concentric contraction of the outer plexiform layer and the centripetal displacement of photoreceptors [27]. Although the fovea was filled with glial tissue [28], atrophy of the photoreceptor layer caused irreversible damage such that the fovea lost its original stratified structure. Hence, it is explicable that the postsurgical BCVA between closed and unclosed MHs was not significantly different.

In the combined group, the recovery of retinal reattachment and MH closure was earlier than that in the MB group. There might be two recovery patterns for the different surgical methods. In the MB group, retinal reattachment occurred due to scleral imbrications that attached the eyeball to the retina, and MH closure was caused by the migration of activated glial cells over time. In combined surgery, the mechanism of MH closure was different, as the inverted ILM flap formed a bridge that provided a scaffold for cells to encroach and enclose the hole. Then, the subretinal fluid was absorbed gradually, and the retina was reattached. Even so, the combined surgery resulted in earlier retinal reattachment and MH closure than MB alone.

In our study, only 69% of patients with combined surgery underwent a successful ILM flap inversion procedure, while the other 31% were unable to complete this procedure because the surgeon could not peel a relatively complete ILM to cover the MH. Due to the unique anatomy of an eye with high myopia and considering its long axial length, thin neurosensory layers, thin ILM and poor contrast, performing procedures in eyes with high myopia are challenging even for an experienced surgeon. Meta-analysis [7] showed that the inverted ILM flap technique could reach a high MH closure ranging from 91.8% to 97.1% and could be an alternative to the treatment of MH in high myopia with or without retinal detachment. In our study, in cases of successful ILM reversal, all the macular holes were closed. Inversion of the ILM flap might have served as a bridge, thus providing scaffolding for tissue proliferation which aids in the healing process of MH. Even though there was no difference in BCVA after surgery between the MH closure and nonclosure groups, MH closure reduced the risk of retinal detachment later in life [29].

Choroidal neovascularization was found to have developed in one case. This might be due to intraoperative and postoperative inflammation that induced the expression of vascular endothelial growth factor. Another reason might be the compression force of the buckle implant disturbing the microcirculatory drainage of the choroid [30], leading to local ischaemia and hypoxia.

Intraretinal cysts were seen in five eyes, which has never been reported in the eyes treated by PPV alone. The presence of cysts was seen as cystoid changes without subretinal fluid. This was likely due to atrophy or loss of tissue. Poor RPE function due to extremely high myopia might be the cause of retinal degeneration and failure of retinal outflow mechanisms, explaining the presence of postsurgery intraretinal cysts.

Our study had an adequate follow-up period of 24 months after surgery, which was a strength of our investigation. However, there were some limitations. First, this was a single-site study, the sample size was relatively small and the loss to follow up was relatively high. Second, such manoeuvres may be technically challenging even for experienced surgeons, as only 69% of patients in the combined group had a successful ILM flap inversion due to an excessively long axis and thin ILM. Third, the mechanisms behind the lack of a significant difference in BCVA between closed and unclosed MHs as well as the development of postsurgical CNV and intraretinal cysts remain unclear, and further research is needed.

In conclusion, MB combined with ILM flap inversion achieved significant BCVA improvement and a higher MH closure rate in high myopia patients with FTMH- associated macular detachment. MH closure could prevent future retinal detachments. Therefore, it is a recommended method of surgical management for MH-associated MD in high myopia patients.

## Summary table

### What was known before

- The macular hole closure rate was relatively low with macular buckling alone, whereas PPV alone often caused more severe macular atrophy progression after surgery which impair the postoperative BCVA.

### What this study adds

- MB combined with PPV and the ILM flap inversion technique achieved better postoperative BCVA and a higher success rate of MH closure than MB alone.

## Supplementary information

suppl table 1

suppl table 2

## Data Availability

The data was available from the corresponding author on the reasonable request.
