# Supplementary material for: Macular buckling alone versus combined inverted ILM flap on macular hole-associated macular detachment in patients with high myopia
Source: Eye (Lond). 2023 Jan 31;37(13):2730–5. doi: 10.1038/s41433-023-02406-1 (PMC10482839; doi:10.1038/s41433-023-02406-1)
Supplement: Supplementary file 1 — suppl table 1 [file 41433_2023_2406_MOESM1_ESM.docx]

Suppl table 1. Comparison of the retinal reattachment and macular hole closure between the MB and the combination group

|  | Retinal reattachment | |  | Macular hole closure | | | | |  | |
| --- | --- | --- | --- | --- | --- | --- | --- | --- | --- | --- |
|  | MB (33) | Combination (29) | *P* | |  | MB (33) | Combination (29) | *P* | |  |
| 1month | 18 (54.6%) | 24 (82.8%) | 0.018 | |  | 6 (18.2%) | 19 (65.5%) | < 0.001 | |  |
| 3-month | 23 (69.7%) | 25 (86.2%) | 0.121 | |  | 13 (39.4%) | 19 (65.5%) | 0.04 | |  |
| 6-month | 26 (78.8%) | 27 (93.1%) | 0.217 | |  | 18 (54.6%) | 21 (72.4%) | 0.234 | |  |
| 12-month | 32 (97.0%) | 29 (100%) | 1.000 | |  | 20 (60.6%) | 22 (75.9%) | 0.313 | |  |
| 24-month | 32 (97.0%) | 29 (100%) | 1.000 | |  | 22 (66.7%) | 24 (82.8%) | 0.248 | |  |
