# Supplementary material for: Macular buckling alone versus combined inverted ILM flap on macular hole-associated macular detachment in patients with high myopia
Source: Eye (Lond). 2023 Jan 31;37(13):2730–5. doi: 10.1038/s41433-023-02406-1 (PMC10482839; doi:10.1038/s41433-023-02406-1)
Supplement: Supplementary file 2 — suppl table 2 [file 41433_2023_2406_MOESM2_ESM.docx]

Suppl table 2. Comparison of the AL in highly myopic eyes with macular hole associated macular detachment between the MB and the combination group

|  | MB | Combination | *P* |
| --- | --- | --- | --- |
| Pre-op | 29.77±1.49 | 29.62±1.90 | 0.734 |
| 6 months | 26.15±2.16 | 26.84±2.34 | 0.229 |
| 12 months | 26.78±2.11 | 27.29±2.42 | 0.381 |
| 24 months | 27.07±2.13 | 27.27±2.41 | 0.499 |
| *P* | < 0.001 | < 0.001 |  |

AL, axial length; MB, macular buckling
